# Digitally planned bimaxillary orthognathic surgery with 3D-printed splints for skeletal class III malocclusion: A case series

**DOI:** 10.1097/MD.0000000000044726

**Published:** 2025-09-26

**Authors:** Viet Anh Nguyen, Truong Minh Nguyen

**Affiliations:** a Faculty of Dentistry, Phenikaa University, Hanoi, Vietnam; b School of Dentistry, Hanoi Medical University, Hanoi, Vietnam.

**Keywords:** 3D-printed surgical splints, bimaxillary orthognathic surgery, digital surgical planning, presurgical orthodontics, skeletal class III malocclusion

## Abstract

**Rationale:**

This retrospective, single-center, non-consecutive case series explores the outcomes of digitally planned bimaxillary orthognathic surgery using three-dimensional (3D) printed surgical splints in the treatment of skeletal class III malocclusion.

**Patient concerns:**

Three adult male patients who underwent combined orthodontic–orthognathic treatment at a private orthodontic center between January 2022 and June 2024 were included nonconsecutively. Presurgical orthodontic preparation was undertaken to decompensate the incisors, align the dental arches, and create optimal conditions for precise skeletal corrections.

**Diagnoses:**

The diagnoses were class III malocclusions on skeletal class III bases with varying vertical facial patterns.

**Interventions:**

Surgical planning incorporated advanced 3D technologies, including multi-slice computed tomography and computer-aided design, to customize surgical movements and fabricate splints via digital light processing 3D printing.

**Outcomes:**

Postoperatively, significant improvements were observed in facial aesthetics, occlusion, and skeletal symmetry. Class I occlusion with aligned dental midlines was achieved in all patients. Although slight reductions in upper airway volume were noted in 2 cases, no functional impairments or obstructive sleep apnea symptoms occurred. The use of 3D-printed splints facilitated accurate surgical execution, particularly in complex movements requiring multi-plane adjustments.

**Lessons:**

This case series highlights the benefits of integrating 3D printing technology with digital surgical planning to enhance precision, efficiency, and clinical outcomes in bimaxillary orthognathic surgery. The findings emphasize the importance of combining advanced fabrication methods with tailored treatment protocols to achieve functional and aesthetic goals in patients with skeletal class III malocclusion.

## 1. Introduction

Skeletal class III malocclusion, characterized by mandibular prognathism and/or maxillary deficiency, is a prevalent dentofacial deformity that poses both functional and aesthetic challenges.^[1]^ Patients with this condition often experience impaired occlusal function, compromised facial harmony, and associated issues such as speech difficulties and respiratory dysfunctions.^[2–4]^ The condition significantly impacts the quality of life, contributing to psychosocial challenges, including diminished self-esteem and social anxiety.^[5]^ Orthognathic surgery, particularly bimaxillary procedures involving Le Fort I osteotomy for maxillary advancement and bilateral sagittal split osteotomy for mandibular setback, has emerged as the definitive treatment for achieving optimal facial and skeletal correction.^[6–8]^

Recent advancements in three-dimensional (3D) imaging and virtual surgical planning have revolutionized the field of orthognathic surgery, offering improved precision, safety, and outcomes. Digital technologies enable surgeons to visualize complex skeletal deformities, simulate surgical interventions, anticipate challenges before the procedure, and design and manufacture surgical splints through subtractive manufacturing or 3D printing. Virtual surgical planning integrates multi-slice computed tomography (MSCT), cone-beam computed tomography, stereophotogrammetry, and computer-aided design to ensure a tailored approach to each patient. Studies highlight that digitally guided orthognathic surgery significantly enhances surgical accuracy, reduces operative time, and improves postoperative skeletal stability.^[6,9,10]^

Despite these advancements, achieving stability and addressing anatomical complexities, such as condylar positioning and airway changes, remain critical challenges. For instance, bimaxillary surgery is associated with potential impacts on the temporomandibular joint, upper airway morphology, and facial soft-tissue contours. Proper alignment of the proximal and distal mandibular segments is essential for preventing relapse and ensuring long-term success.^[2,10,11]^ Moreover, changes in oral cavity volume and upper airway dimensions, commonly observed postoperatively, necessitate careful preoperative planning to minimize adverse effects.^[2,4]^

Equally important are the psychological benefits of orthognathic surgery. Studies demonstrate that correcting skeletal class III malocclusion not only improves functional outcomes but also enhances self-esteem, reduces sensitivity to criticism, and alleviates social anxiety, underscoring the profound life-changing effects of such interventions.^[3,5]^

This case series investigates the outcomes of digitally planned bimaxillary orthognathic surgery in treating skeletal class III malocclusion. By focusing on skeletal stability, airway and condylar adaptations, and patient-reported outcomes, this report aims to provide a comprehensive evaluation of the efficacy and precision of digital planning in managing complex dentofacial deformities.

## 2. Patients and methods

This retrospective, single-center, nonconsecutive case series was conducted at a private orthodontic center in Hanoi. All electronic records of adults treated with combined orthodontic–orthognathic therapy at a private orthodontic center between January 2022 and June 2024 were screened. Inclusion criteria were skeletal class III requiring bimaxillary surgery, complete pre- and posttreatment cephalometric radiographs, and no craniofacial syndromes or prior orthognathic procedures. Exclusion criteria were incomplete imaging, previous jaw surgery, or syndromic conditions. Three nonconsecutive male cases meeting these criteria were purposefully selected to represent hypodivergent, normodivergent, and hyperdivergent vertical patterns for illustrative comparison.

Presurgical orthodontic preparation decompensated incisors, aligned and leveled both arches, and standardized maxilla-first bimaxillary surgery was performed by a maxillofacial surgeon using a uniform digital workflow, with splints printed in identical resin on a single digital light processing (DLP) printer. Virtual planning was performed in ProPlan CMF (Materialise NV, Leuven, Belgium), a commercially available platform accessible to licensed clinicians and institutions. Effective use requires competency in image acquisition, segmentation, orientation of reference planes, dental model alignment, and splint design. Surgeon experience materially influences plan quality, particularly in landmark placement, occlusal plane definition, condylar seating decisions, and anticipating soft-tissue or airway effects. In our center, all cases were planned by the same surgeon after dedicated training to standardize the workflow.

All patients underwent bimaxillary orthognathic surgery, including Le Fort I advancement and bilateral sagittal split osteotomy, with genioplasty performed when indicated. A standardized peri-operative medication protocol was implemented, consisting of intravenous cefuroxime 1.5 g twice daily, intravenous metronidazole 500 mg/100 mL 3 times daily, intravenous methylprednisolone 40 mg once each morning, and intravenous esomeprazole 40 mg once daily. Analgesia comprised intravenous paracetamol 1 g every 12 hours. Intravenous fluids included 5% glucose 500 mL and lactated Ringer 500 mL as clinically indicated. Preventive measures included meticulous intraoperative asepsis, while pharmacologic prophylaxis was reserved for elevated-risk profiles.

Postoperatively, patients were instructed to maintain head elevation for 48 to 72 hours, apply intermittent cold packs for the first 24 to 48 hours, and adhere to meticulous oral hygiene with a soft toothbrush plus 0.12% chlorhexidine mouthrinse. A soft, non-chew diet was prescribed for 4 to 6 weeks, with progressive return to normal consistency thereafter. Where guiding elastics were used, wear was full-time except during oral hygiene, according to weekly adjustments. Targeted jaw physiotherapy (passive and then active range-of-motion exercises) commenced between postoperative days 7 to 14 depending on comfort, aiming for gradual increases in interincisal opening, lateral, and protrusive excursions. Follow-up occurred at 1 week, 2 weeks, and monthly thereafter during the finishing orthodontic phase.

For outcome measurement, 2 calibrated investigators independently traced the cephalometric landmarks, achieving an intraclass correlation coefficient of 0.936. The study protocol was reviewed and approved by the Institutional Review Board of Phenikaa University (approval no. 024-03.04, December 20, 2024). Owing to the retrospective design of this study, the requirement for written informed consent for participation was waived by the board. However, all patients provided written informed consent for the use of their clinical data and images for publication purposes.

## 3. Case 1 presentation

**Figure 1.**
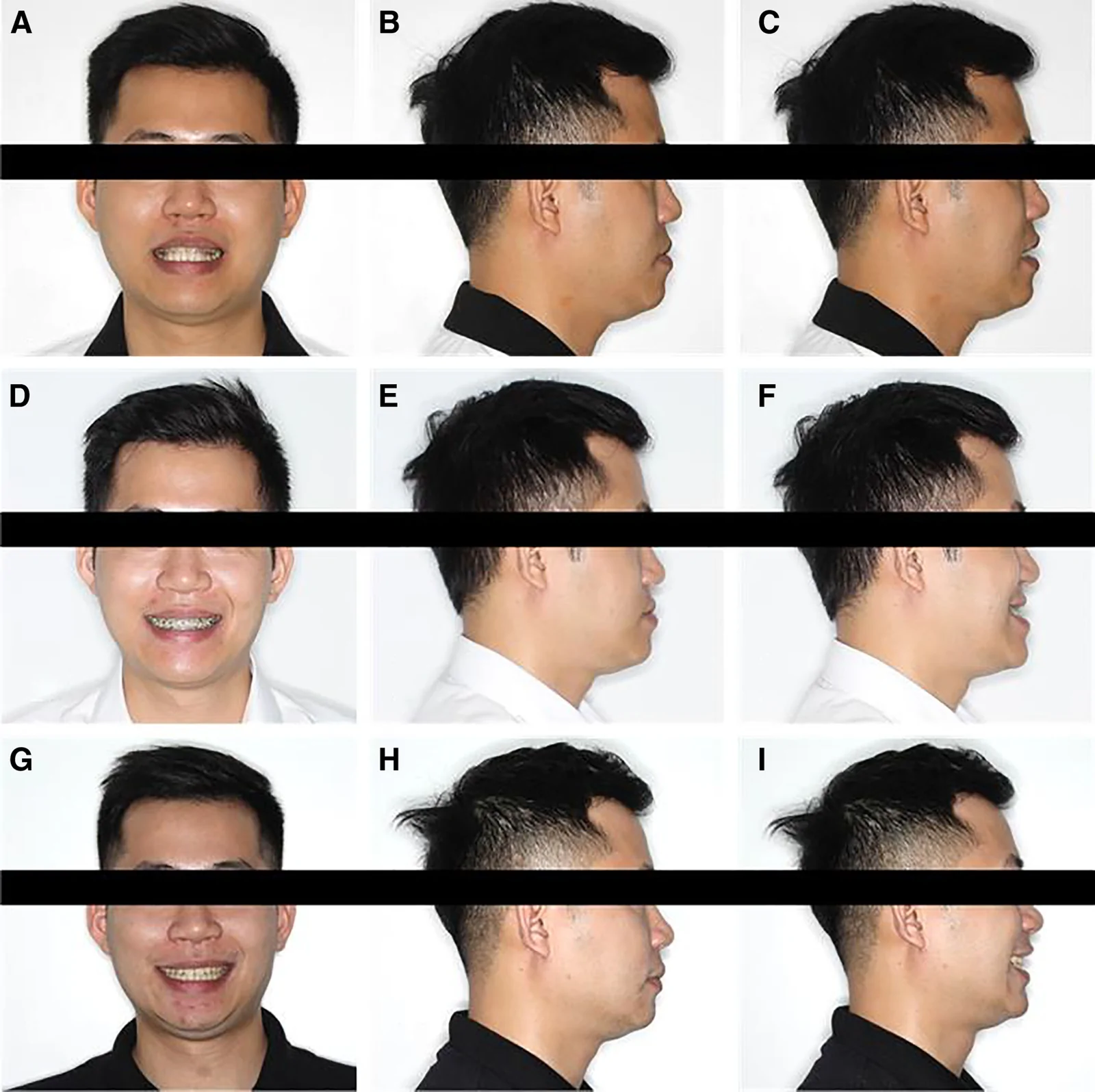
Case 1, facial photographs. (A–C) Pretreatment. (D–F) Postsurgery. (G–I) Posttreatment.

**Figure 2.**
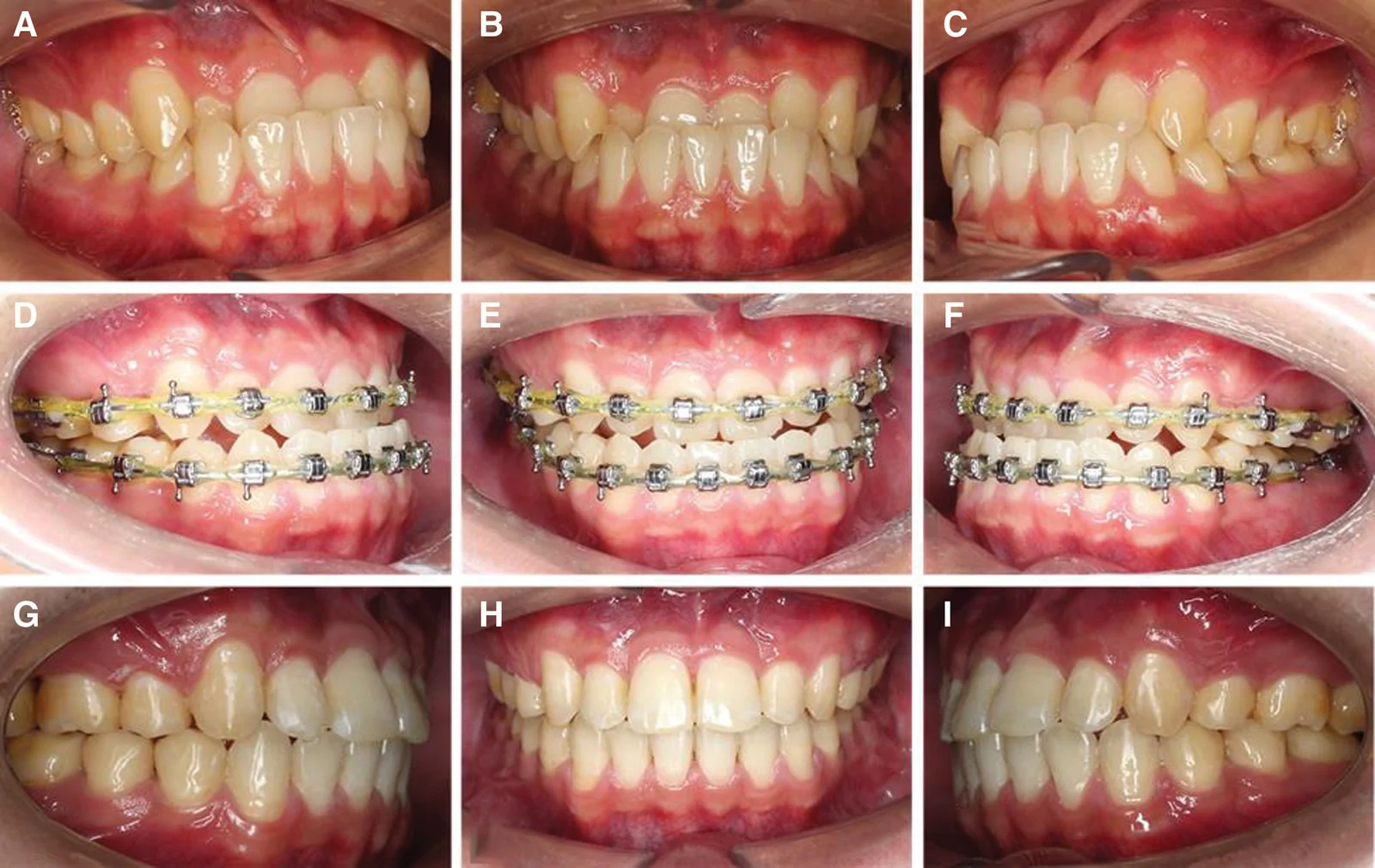
Case 1, occlusion photographs. (A–C) Pretreatment. (D–F) Postsurgery. (G–I) Posttreatment.

**Figure 3.**
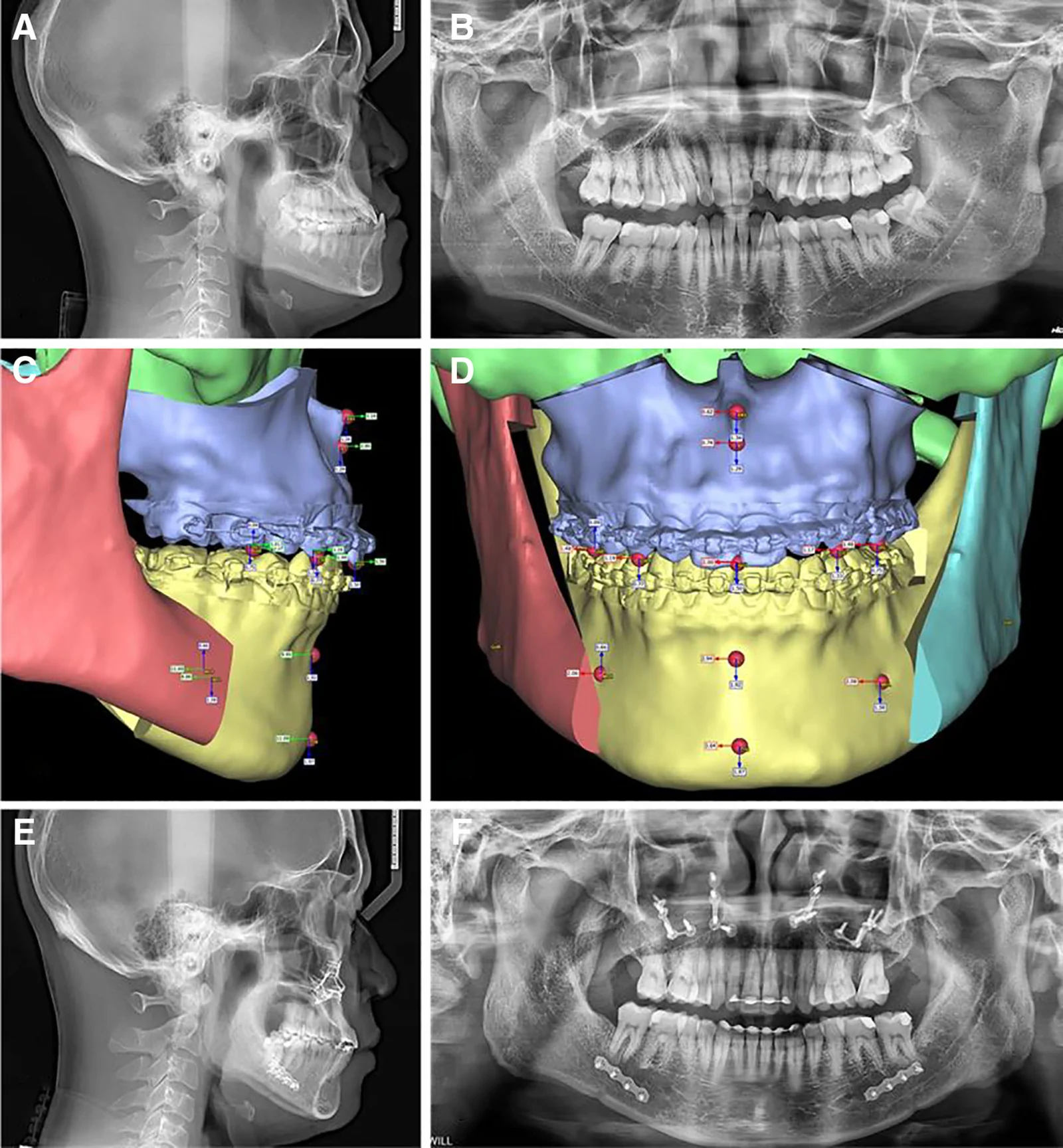
Case 1, radiographs. (A, B) Pretreatment. (C, D) Surgical plan. (E, F) Posttreatment.

A 27-year-old male patient presented with concerns about an underbite and a prominent chin, which significantly impacted his facial aesthetics and functional comfort. Upon extraoral examination, the patient exhibited a concave profile, reduced lower anterior facial height, and a left-deviated mandible (Fig. 1A–C). Intraorally, moderate crowding was noted in the upper arch, along with mild crowding in the lower arch and a class III dental relationship (Fig. 2A–C). The upper incisors exhibited slight labial inclination, while the lower incisors were inclined lingually (Fig. 3A, B). The patient was diagnosed with a class III malocclusion on a hypodivergent skeletal class III base due to a prognathic mandible. Given the moderate upper arch crowding, the treatment plan included extraction of the upper first bicuspids to decompensate the upper incisors and alleviate crowding, in conjunction with bimaxillary osteotomy to address the skeletal discrepancies. A traditional orthognathic approach was chosen over a surgery-first protocol to allow for precise preoperative orthodontic alignment and occlusal planning. Orthodontic treatment commenced with the goal of aligning the teeth, closing the extraction spaces in the upper arch, and creating a negative overjet to facilitate proper skeletal realignment during surgery. Over a 13-month period, the teeth in both arches were aligned, and the upper extraction spaces were closed (Fig. 1D–F). By the end of this phase, a pronounced negative overjet was established, optimizing the conditions for surgical repositioning of the maxillomandibular complex (Fig. 2D–F). The surgical plan was meticulously developed utilizing intraoral scans and MSCT data, processed through ProPlan CMF digital planning software. The proposed surgical movements included maxillary advancement of 2.8 mm, mandibular setback of 9.9 mm, and a clockwise rotation of both jaws to increase the lower anterior facial height (Fig. 3C, D). Surgical splints were fabricated using a DLP 3D printer (Photon D2, Anycubic, Shenzhen, China) with photosensitive resin (Surgical Guide, Ludent, Gyeonggi-do, Korea) to ensure precise jaw placement during fixation. The operative sequence involved performing the maxillary osteotomy first, followed by the mandibular osteotomy. The surgery was performed without complications, and intermaxillary fixation was removed immediately postoperatively to allow for condylar adaptation to centric relation and the resumption of normal chewing function. The healing process was uneventful. Orthodontic detailing began 2 weeks postsurgery and continued for an additional 4 months to refine the occlusion. Posttreatment results demonstrated a significantly improved profile with harmonious chin projection and correction of the mandibular deviation (Fig. 1G–I). The patient achieved a class I occlusion bilaterally with stable and functional results (Fig. 2G–I). Postoperative assessments indicated a slight reduction in upper airway dimensions, however, there were no clinical signs or symptoms of obstructive sleep apnea (Fig. 3E, F). The patient expressed high satisfaction with the treatment outcomes, which met both aesthetic and functional goals, including a balanced facial profile and normal occlusal function.

## 4. Case 2 presentation

**Figure 4.**
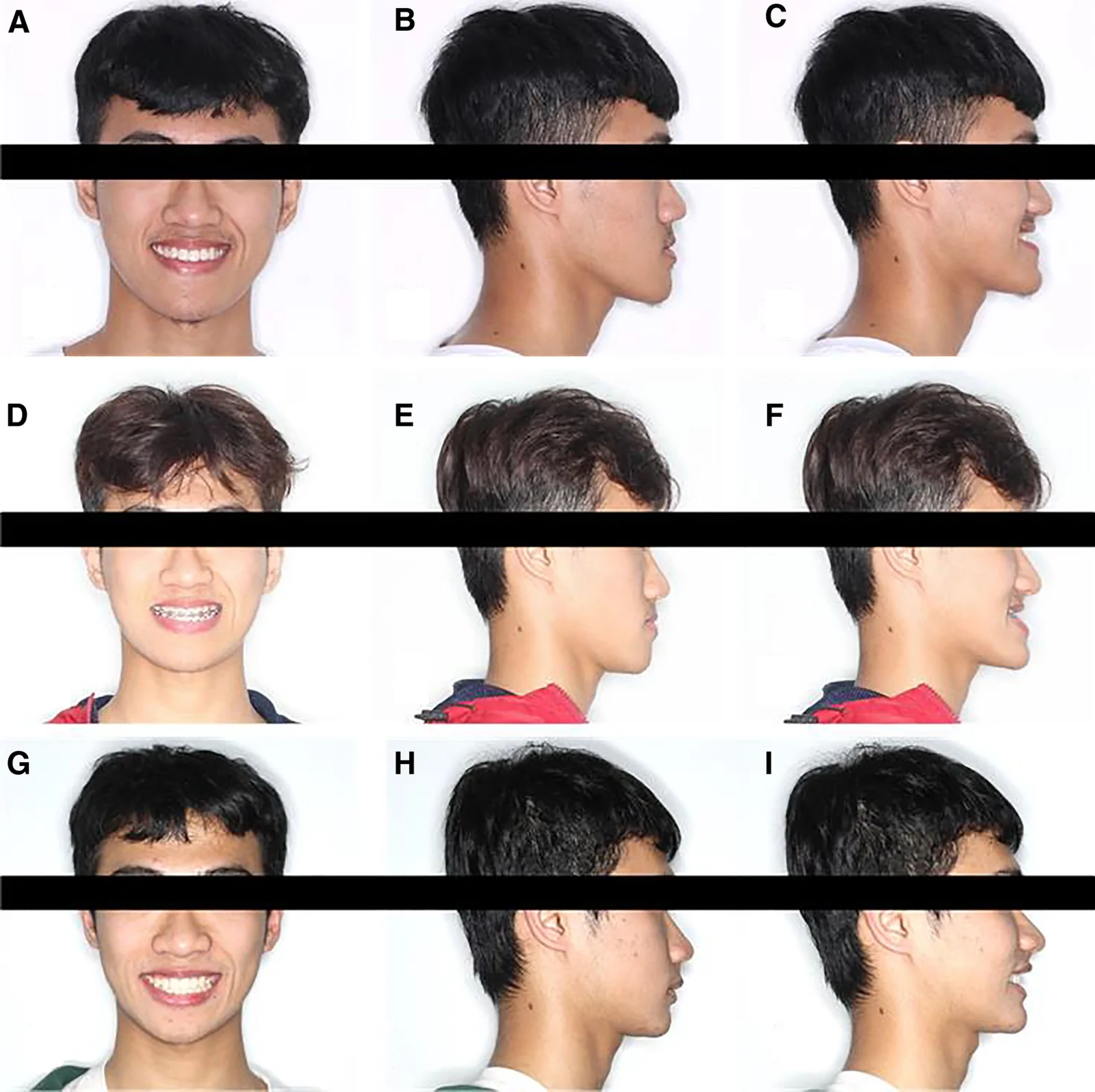
Case 2, facial photographs. (A–C) Pretreatment. (D–F) Postsurgery. (G–I) Posttreatment.

**Figure 5.**
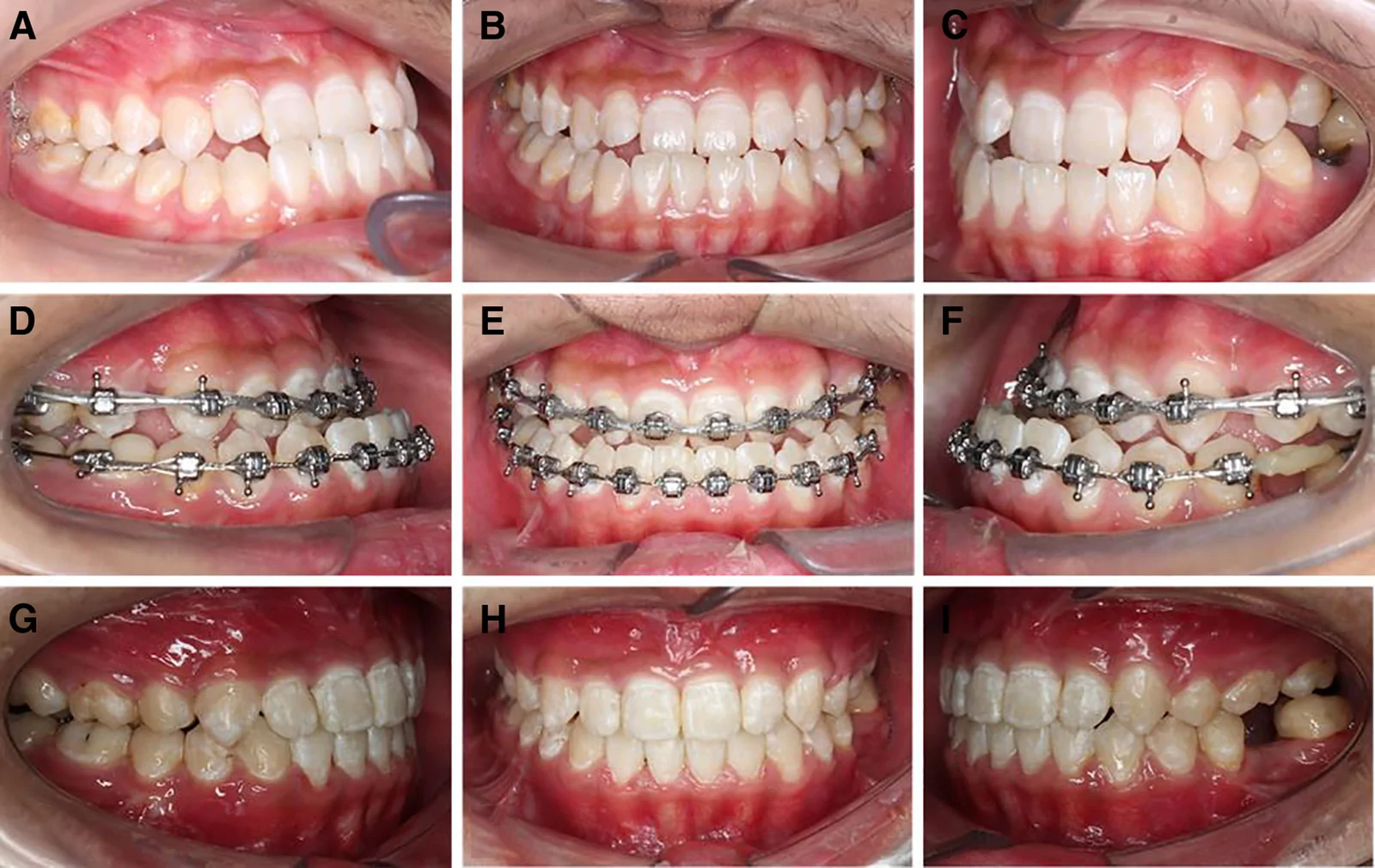
Case 2, occlusion photographs. (A–C) Pretreatment. (D–F) Postsurgery. (G–I) Posttreatment.

**Figure 6.**
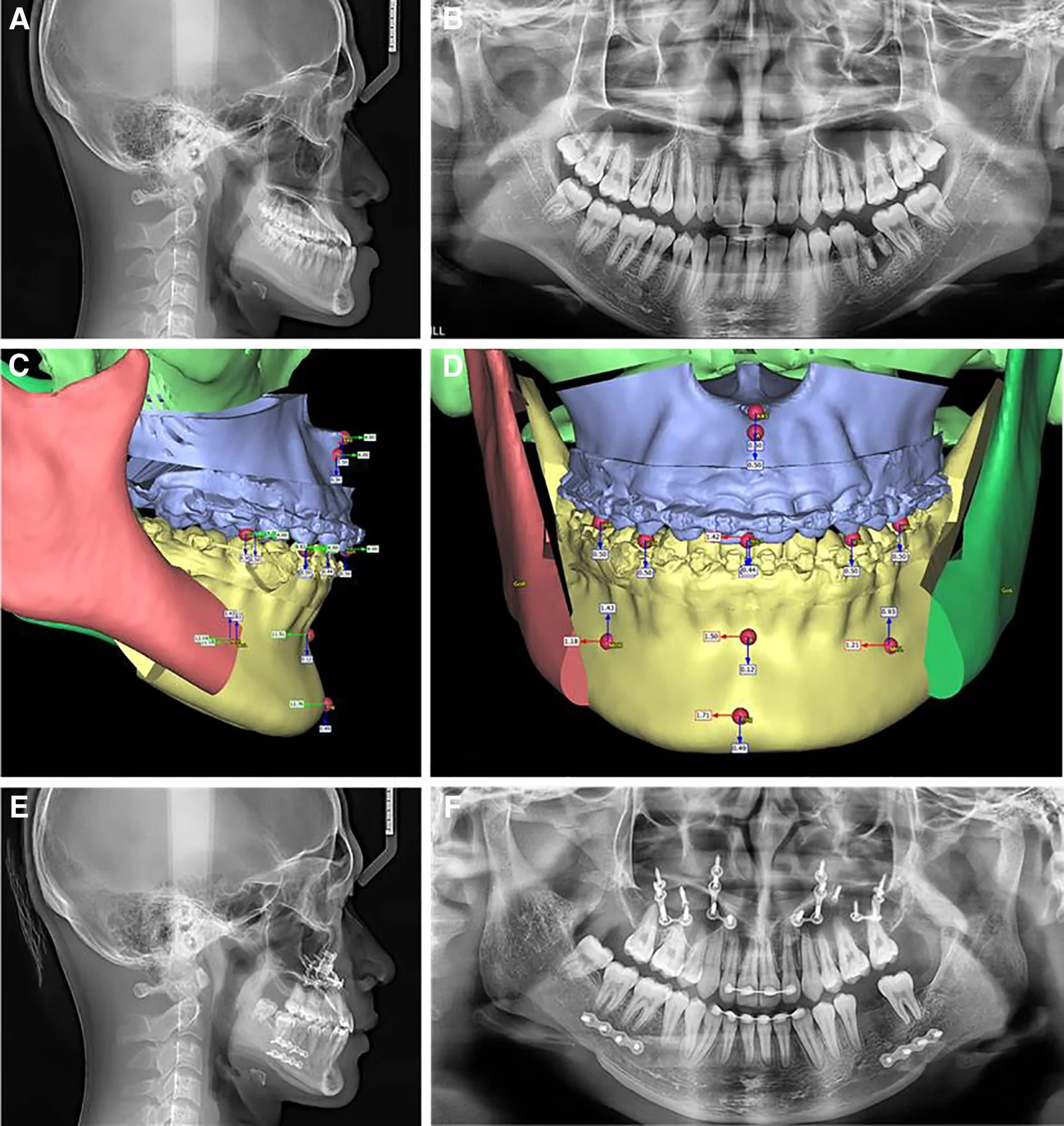
Case 2, radiographs. (A, B) Pretreatment. (C, D) Surgical plan. (E, F) Posttreatment.

An 18-year-old male patient presented with concerns of an underbite and a prominent mandible. On extraoral examination, the patient displayed a concave profile, normal lower anterior facial height, and an elongated mandible (Fig. 4A–C). Intraorally, the patient exhibited a class III occlusion with mild crowding in both arches and an unrestorable lower left first molar (Fig. 5A–C). Significant dental compensation was observed, with the upper incisors proclined and the lower incisors retroclined (Fig. 6A, B). The diagnosis was a class III malocclusion on a normodivergent skeletal class III base due to a prognathic mandible. Given the pronounced proclination of the upper incisors, the treatment plan included extraction of the upper first bicuspids to facilitate incisor decompensation and alignment with the maxillary base. This was combined with bimaxillary osteotomies to address the underlying skeletal discrepancy. A 12-month presurgical orthodontic phase was undertaken to close the upper extraction spaces and decompensate the upper and lower incisors (Fig. 4D–F). By the end of this phase, the incisor inclinations were significantly improved, achieving a negative overjet to optimize conditions for anteroposterior jaw correction during surgery (Fig. 5D–F). The surgical plan was developed using digital planning tools, incorporating a 4.0-mm maxillary advancement and an 11.5-mm mandibular setback (Fig. 6C, D). Custom surgical splints were fabricated using 3D printing to ensure precise intraoperative jaw positioning. The surgery proceeded as planned, and intermaxillary fixation was removed immediately afterward to promote functional adaptation of the condyles. Postoperative healing was uneventful. The postsurgical orthodontic phase lasted 7 months and focused on refining the occlusion. The final results revealed a dramatic transformation in the patient’s facial profile, achieving a harmonious balance between the nose, lips, and chin (Fig. 4G–I). The occlusion was corrected to a bilateral class I relationship with coincident dental midlines (Fig. 5G–I). Posttreatment assessments confirmed no clinical signs of obstructive sleep apnea, despite the surgical alterations (Fig. 6E, F). The patient expressed high satisfaction with the functional and aesthetic outcomes, which successfully addressed his initial concerns.

## 5. Case 3 presentation

**Figure 7.**
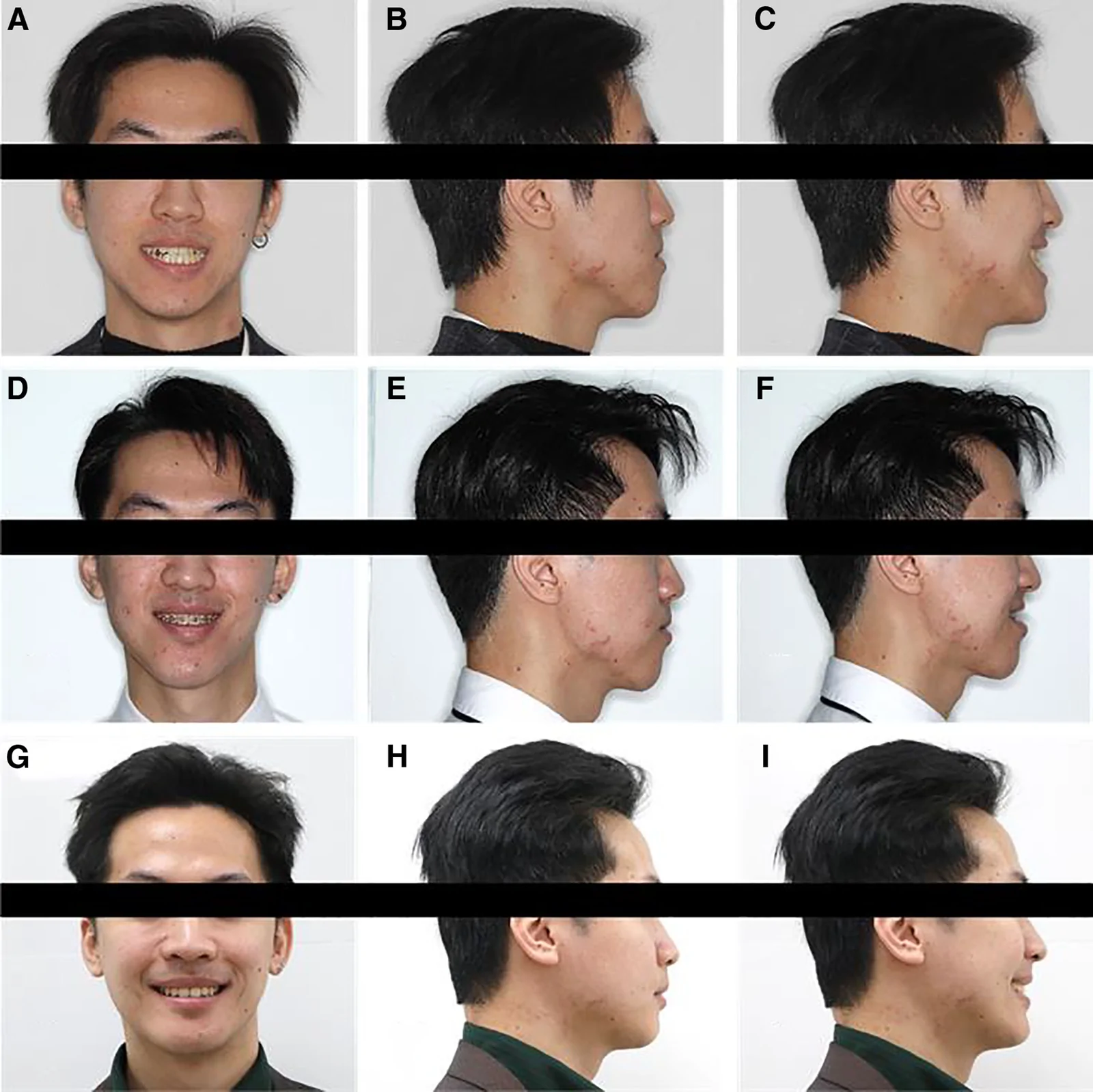
Case 3, facial photographs. (A–C) Pretreatment. (D–F) Postsurgery. (G–I) Posttreatment.

**Figure 8.**
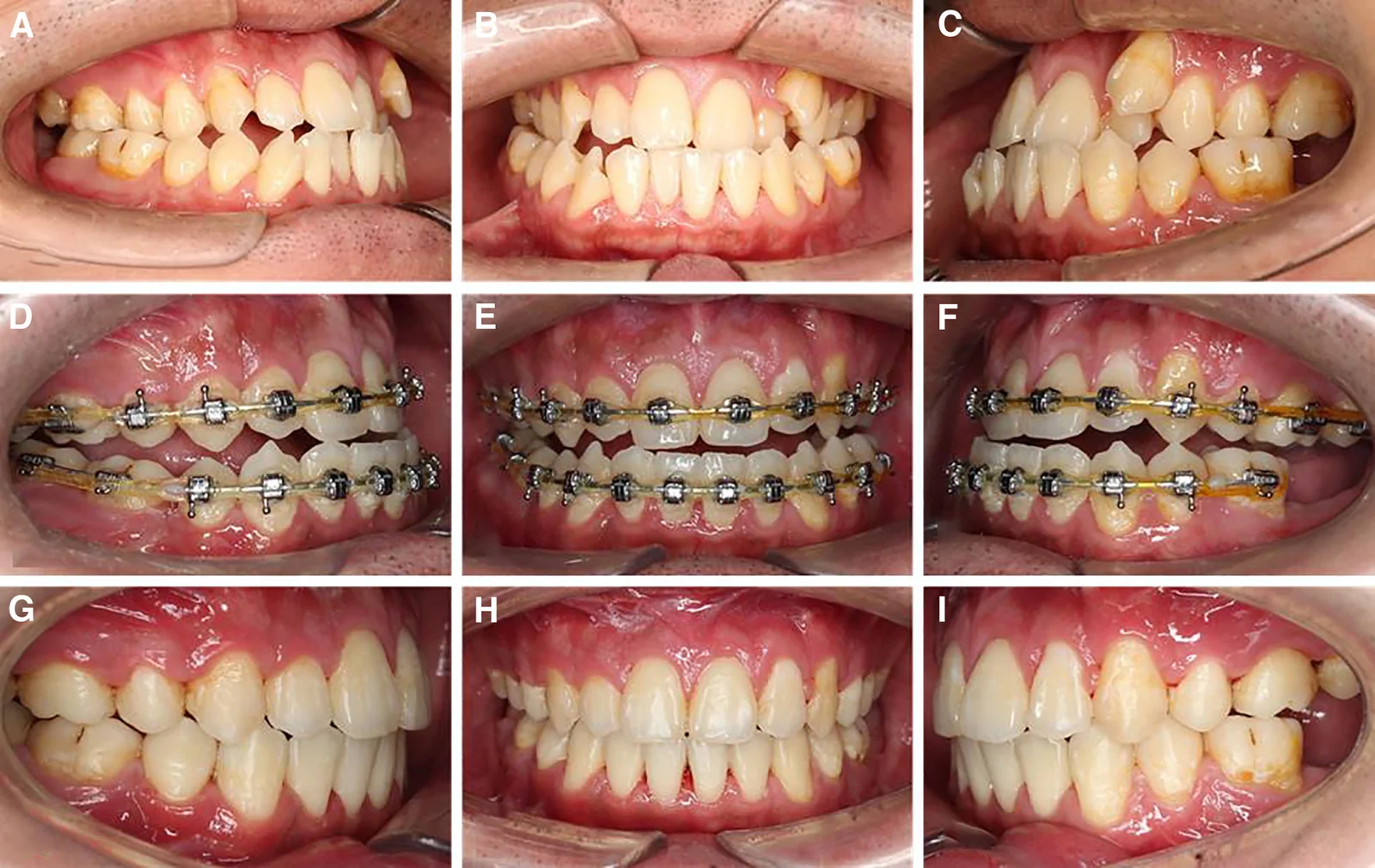
Case 3, occlusion photographs. (A–C) Pretreatment. (D–F) Postsurgery. (G–I) Posttreatment.

**Figure 9.**
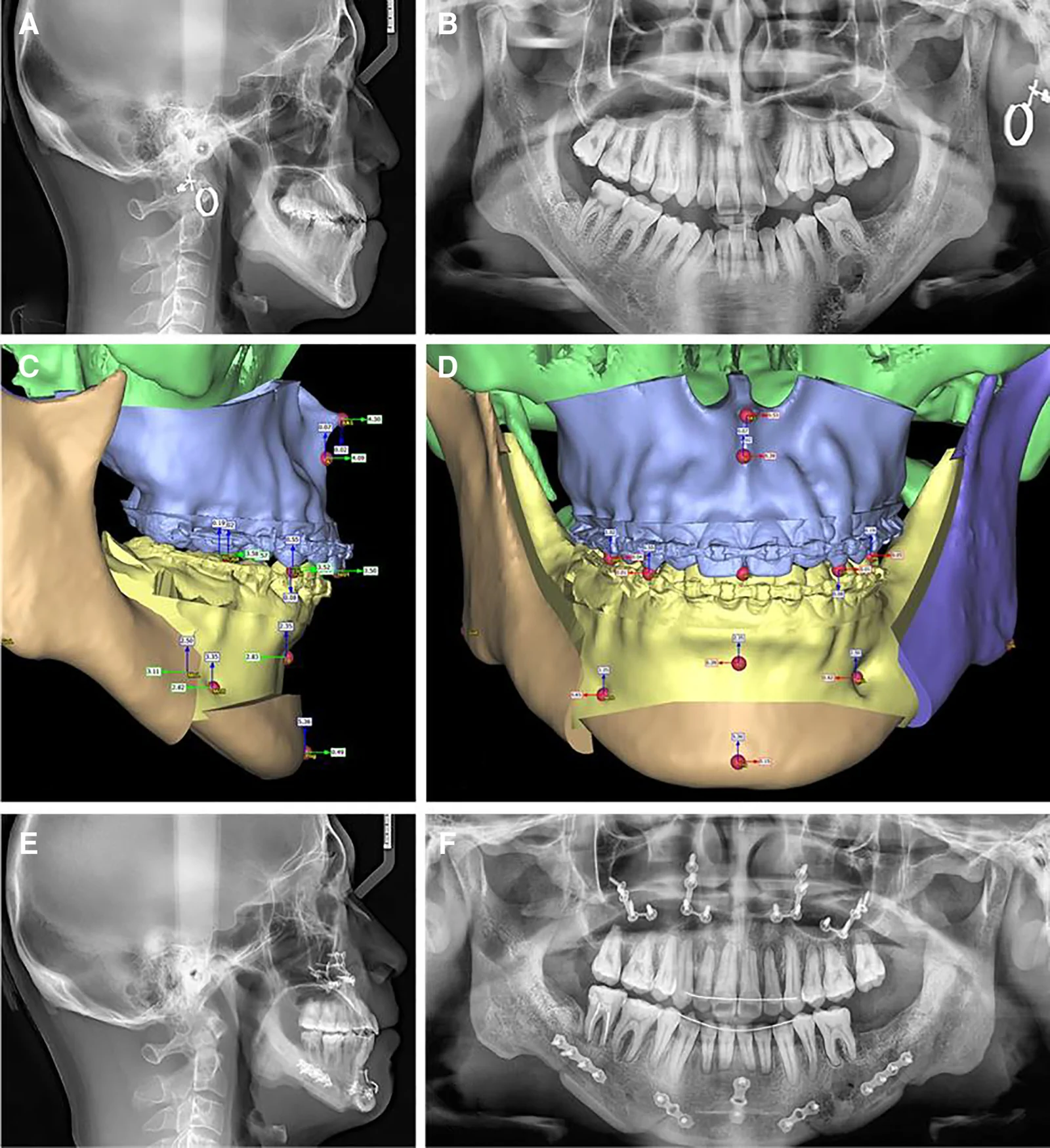
Case 3, radiographs. (A, B) Pretreatment. (C, D) Surgical plan. (E, F) Posttreatment.

A 31-year-old male patient presented with primary concerns of anterior crossbite and crowded teeth, which affected both function and aesthetics. On extraoral examination, the patient displayed a straight profile with prominent lower lips, an absent labiomental sulcus, a right-deviated mandible, and increased lower anterior facial height (Fig. 7A–C). Intraoral findings included severe crowding in the upper arch, moderate crowding in the lower arch, a class III dental relationship, and the absence of the lower left second molar and second bicuspid (Fig. 8A–C). The upper incisors exhibited normal inclination, while the lower incisors were lingually tipped (Fig. 9A, B). The diagnosis was a class III malocclusion on a hyperdivergent skeletal class III base, primarily due to a retruded maxilla. Considering the significant crowding, the treatment plan involved extraction of the upper first bicuspids and the lower right second bicuspid to create space for alignment. This was combined with bimaxillary osteotomies to correct the skeletal hyperdivergence and class III relationship. The presurgical orthodontic phase spanned 18 months, during which the extraction spaces were closed, and alignment of the dental arches was achieved (Fig. 7D–F). By the end of this phase, a negative overjet and slight open bite had been established to facilitate the planned surgical movements (Fig. 8D–F). The orthognathic surgical plan was developed using digital workflows, integrating intraoral scans and MSCT data. The plan involved a maxillary advancement of 4.1 mm, a mandibular setback of 2.8 mm, and a genioplasty involving superior movement of 3 mm combined with an advancement of 3.5 mm (Fig. 9C, D). The surgery proceeded as planned, using digitally designed and 3D-printed surgical splints as guidance during skeletal fixation. Following surgery, a 5-month orthodontic detailing phase was undertaken to refine the occlusion. The final results demonstrated a marked improvement in facial harmony, with better lip balance, chin projection, and a well-defined labiomental sulcus (Fig. 7G–I). Occlusion was corrected to a bilateral class I relationship, with centered dental midlines and well-aligned teeth (Fig. 8G–I). Postoperative imaging confirmed preservation of the upper airway volume, with no signs of obstructive sleep apnea (Fig. 9E, F). The patient expressed high satisfaction with both the functional and aesthetic outcomes, achieving the treatment objectives successfully.

## 6. Results

Three adult male patients with skeletal class III malocclusion representing hypodivergent (case 1), normodivergent (case 2), and hyperdivergent (case 3) vertical patterns completed combined orthodontic–orthognathic treatment. Presurgical orthodontics lasted 13, 12, and 18 months, respectively, to achieve alignment and decompensation. All patients underwent digitally planned bimaxillary surgery using 3D-printed splints. Planned surgical movements varied across the 3 cases (Table 1). All operations proceeded uneventfully, and intermaxillary fixation was removed immediately after surgery.

**Table 1:** Case-level summary of outcomes.

Item	Case 1	Case 2	Case 3
Age, sex	27, male	18, male	31, male
Vertical pattern	Hypodivergent	Normodivergent	Hyperdivergent
Presurgical orthodontics	13 mo	12 mo	18 mo
Extractions performed	Maxillary first premolars	Maxillary first premolars	Maxillary first premolars; mandibular right second premolar
Planned surgical movements	Maxilla +2.8 mm; mandible −9.9 mm; clockwise rotation	Maxilla +4.0 mm; mandible −11.5 mm	Maxilla +4.1 mm; mandible −2.8 mm; genioplasty +3.0 mm superior and +3.5 mm advance
Intraoperative course	Uneventful; intermaxillary fixation removed immediately	Uneventful; intermaxillary fixation removed immediately	Uneventful; intermaxillary fixation removed immediately
Post-op orthodontic detailing	4 mo	7 mo	5 mo
Final occlusion	Bilateral class I; midlines aligned	Bilateral class I; midlines aligned	Bilateral class I; midlines aligned
Facial outcome highlights	Corrected mandibular deviation; improved chin harmony	Harmonized facial profile	Improved lip balance and labiomental sulcus
Airway observation	Slight reduction; asymptomatic	Slight reduction; asymptomatic	Preserved; asymptomatic
Complications	None observed	None observed	None observed
Patient satisfaction	High	High	High

All 3 patients finished with a bilateral class I occlusion and coincident dental midlines. Facial profile and symmetry improved in every case: correction of mandibular deviation and chin-profile harmony in case 1; balanced profile in case 2; enhanced lip balance and labiomental sulcus definition in case 3. Slight postoperative reductions in upper airway dimensions were observed in cases 1 and 2, while case 3 demonstrated preservation of airway volume. No patient-reported symptoms suggestive of obstructive sleep apnea during follow-up. No intra- or postoperative complications were recorded. All patients expressed high satisfaction with both functional and aesthetic outcomes at the end of treatment.

## 7. Discussion

This case series highlights critical aspects of digitally planned bimaxillary orthognathic surgery for treating skeletal class III malocclusion. The outcomes and techniques discussed demonstrate how digital tools and treatment protocols can enhance precision and address the unique anatomical and aesthetic needs of patients.

Three-dimensional-printed surgical splints have revolutionized orthognathic surgery by providing precise, customizable, and efficient tools for transferring digital plans into clinical practice. Compared to conventional methods, 3D printing reduces fabrication errors and enables greater adaptability to complex surgical movements, such as maxillary impaction or multiplane adjustments.^[12,13]^ Techniques like DLP and stereolithography achieve clinically acceptable accuracy, with DLP splints often offering superior clinical fit due to their adaptability.^[14]^ Factors such as post-processing rinsing solvents and polymerization times further influence the accuracy of 3D-printed splints, with isopropyl alcohol rinsing and a 6-minute polymerization time yielding the most precise results.^[15]^ Moreover, advancements in computer-aided design software enable the integration of bone-supported splints, enhancing stability and reducing errors in vertical and transverse movements, especially in complex cases.^[16]^

One of the notable findings is that despite a slight decrease in upper airway volume observed in Cases 1 and 2, there were no functional impairments or clinical signs of obstructive sleep apnea, reflecting preserved airway functionality. While mandibular setback surgery alone is often associated with a significant reduction in airway dimensions, bimaxillary surgery tends to mitigate this effect by allowing adjustments to both the maxilla and mandible. This dual adjustment distributes changes across the skeletal structure, reducing the likelihood of airway compromise.^[2,4]^ These findings align with previous research emphasizing the advantages of bimaxillary surgery in maintaining functional outcomes, particularly in patients with class III malocclusion.^[4,17]^

The relatively smaller maxillary advancement compared to mandibular setback in these cases reflects a conscious effort to align with Asian aesthetic preferences. Studies have shown that Asian individuals generally favor slightly retruded lips for an ideal profile.^[18,19]^ These cultural preferences influence treatment planning, highlighting the importance of tailoring surgical interventions to the patient’s ethnic background and societal norms. The 3D planning tools allowed precise customization of the maxillomandibular relationship, ensuring both functional improvement and aesthetic harmony.

All 3 cases adopted a maxilla-first approach during surgery. While the maxilla-first sequence is traditionally preferred in cases involving maxillary posterior impaction or clockwise rotation, recent evidence suggests that the mandible-first sequence can achieve comparable surgical accuracy and stability in skeletal class III malocclusion.^[20]^ A study concluded that the discrepancies between planned and actual outcomes were not significantly different between the 2 sequences, even when mandibular autorotation was required for intermediate splint application.^[21]^ This finding indicates that the choice of surgical sequence can be based on the specific clinical situation and surgeon’s preference without compromising the precision of the outcomes.

Two clinical questions emerged from this case series. First, how best to balance mandibular setback magnitude with airway preservation in class III patients? Although no clinical obstructive sleep apnea was observed here, the relatively large setbacks in some cases suggest favoring greater maxillary advancement or counterclockwise rotation when feasible to limit posterior tongue displacement. Second, when should a mandible-first sequence be preferred? For cases dominated by asymmetry or when maxillary movements are limited, a mandible-first approach may simplify intraoperative seating and reduce reliance on intermediate splints. In future similar cases, we plan to incorporate preoperative airway risk stratification with selective sleep studies in at-risk patients, prioritize surgical plans that distribute correction between the jaws to minimize extreme setbacks, and consider bone-supported or navigation-assisted guides for large vertical or transverse corrections.

The use of presurgical orthodontic phases in all cases was essential for achieving decompensation of the upper and lower incisors, aligning dental arches, and creating conditions conducive to accurate surgical corrections.^[22]^ While surgery-first protocols are gaining popularity, presurgical orthodontics remains critical in cases with severe crowding or significant dental compensation. This preparatory phase facilitates precise skeletal movements during surgery and improves the predictability of postoperative outcomes.^[10]^

This retrospective, single-center, nonconsecutive case series included a small, all-male sample without a control group, limiting generalizability. Follow-up was limited to the orthodontic finishing period; longer observation is required to assess stability and airway adaptation. Airway assessment was qualitative and lacked polysomnography or standardized patient-reported outcome measures. Printing accuracy and material properties may vary across devices and post-processing protocols, and our workflow used a single printer and resin. Future work should include prospective cohorts with larger, balanced samples; quantitative airway and sleep assessments; and learning-curve studies comparing maxilla-first and mandible-first sequences.

## 8. Conclusion

This case series emphasizes the advantages of digitally planned bimaxillary orthognathic surgery with 3D-printed splints in achieving functional, aesthetic, and stable outcomes for skeletal class III malocclusion. By integrating a presurgical orthodontic phase, advanced imaging, tailored surgical planning, and precise 3D printing technology, this approach effectively addresses anatomical complexities, enhances surgical precision, and aligns with cultural aesthetic preferences, offering comprehensive and reliable care for patients.

## Author contributions

**Conceptualization:** Viet Anh Nguyen.

**Formal analysis:** Viet Anh Nguyen.

**Investigation:** Viet Anh Nguyen.

**Methodology:** Viet Anh Nguyen, Truong Minh Nguyen.

**Project administration:** Viet Anh Nguyen.

**Software:** Truong Minh Nguyen.

**Supervision:** Viet Anh Nguyen.

**Visualization:** Truong Minh Nguyen.

**Writing – original draft:** Viet Anh Nguyen.

**Writing – review & editing:** Viet Anh Nguyen.
